# Associations of Brachial-Ankle Pulse Wave Velocity With Left Ventricular Geometry and Diastolic Function in Untreated Hypertensive Patients

**DOI:** 10.3389/fcvm.2021.647491

**Published:** 2021-05-10

**Authors:** Soongu Kwak, Hack-Lyoung Kim, Minjae In, Woo-Hyun Lim, Jae-Bin Seo, Sang-Hyun Kim, Joo-Hee Zo, Myung-A Kim

**Affiliations:** ^1^Division of Cardiology, Department of Internal Medicine, Boramae Medical Center, Seoul National University College of Medicine, Seoul, South Korea; ^2^Seoul National University College of Medicine, Seoul, South Korea

**Keywords:** arterial stiffness, diastolic function, hypertension, left ventricular remodeling, pulse wave velocity

## Abstract

**Background:** Although brachial-ankle pulse wave velocity (baPWV) is simple and convenient, its usefulness as an initial screening test for hypertensive patients is not well-known. This study aimed to investigate the association of baPWV with left ventricular (LV) geometry and diastolic function in treatment-naive hypertensive patients.

**Methods:** A total of 202 untreated hypertensive patients (mean age, 62 years; males, 51.5%) without documented cardiovascular diseases were prospectively enrolled. Both baPWV and transthoracic echocardiography were performed on the same day before antihypertensive treatment.

**Results:** In multiple linear regression analysis after adjustment for potential confounders, baPWV had significant correlations with structural measurements of LV including relative wall thickness (β = 0.219, *P* = 0.021) and LV mass index (β = 0.286, *P* = 0.002), and four diastolic parameters including septal e′ velocity (β = −0.199, *P* = 0.018), E/e′ (β = 0.373, *P* < 0.001), left atrial volume index (β = 0.334, *P* < 0.001), and maximal velocity of tricuspid regurgitation (β = 0.401, *P* < 0.001). The baPWV was significantly increased in patients with LV hypertrophy, abnormal LV remodeling, or diastolic dysfunction, compared to those without (*P* = 0.008, *P* = 0.035, and *P* < 0.001, respectively). In the receiver operating characteristic curve analysis, the discriminant ability of baPWV in predicting LV hypertrophy and diastolic dysfunction had an area under the curve of 0.646 (95% confidence interval 0.544–0.703, *P* = 0.004) and 0.734 (95% confidence interval 0.648–0.800, *P* < 0.001), respectively.

**Conclusion:** baPWV was associated with parameters of LV remodeling and diastolic function in untreated hypertensive patients. The baPWV could be a useful screening tool for the early detection of adverse cardiac features among untreated hypertensive patients.

## Introduction

Increased arterial stiffness reflects the vascular damage and arteriosclerosis (1, 2), and it is associated with a higher risk of cardiovascular events and mortality (3–8). The worse prognosis related to increased arterial stiffness is supported by the fact that arterial stiffening has a significant association with an abnormal structural remodeling of the left ventricle (LV) (9–11), impairment of contractile performance (9, 12), and diastolic dysfunction (10, 11, 13–19), all of which are key mechanisms in the development of heart failure. Among the several methods for quantifying arterial stiffness, pulse wave velocity (PWV), which is defined as the propagation velocity of pressure wave produced by heartbeat, is the most widely used (2, 20). Although the guideline suggests the assessment of carotid-femoral PWV (cfPWV) for detecting vascular damage in hypertensive patients (21), brachial-ankle PWV (baPWV) is simpler, less time-consuming, and more convenient method for both examiners and patients (20, 22–24), which may hold the potential in generalizing its usage in practice. This study aimed to examine the associations of baPWV with LV geometry and diastolic function in untreated hypertensive patients and to provide support for its clinical value in the assessment of target organ damages in the initial evaluation of hypertension.

## Materials and Methods

### Study Population

This prospective cohort study was conducted at a general hospital in a big city (Seoul, South Korea). Patients who visited or were referred to the outpatient clinic to evaluate hypertension for the first time and not previously diagnosed with hypertension nor receiving antihypertensive medications were consecutively recruited between December 2016 and October 2020. The diagnosis of hypertension was made when the systolic/diastolic blood pressure of ≥140/90 mmHg was confirmed at least twice in the office. The office blood pressure measurement was performed by a single experienced cardiologist (H.L.K) using the oscillometric method on the right upper arm. If the diagnosis of hypertension was not determined through the office blood pressure measurements, 24-h ambulatory blood pressure monitoring was additionally performed with the diagnostic cutoff of systolic/diastolic blood pressure of ≥135/85 mmHg (21). Hypertension mediated organ damages (HMODs) were assessed shortly after the hypertension diagnosis, and all patients underwent baPWV measurement and transthoracic echocardiography on the same day. Screening for the secondary causes of hypertension was also investigated under the suggestive symptoms, clinical signs, and abnormal laboratory or imaging tests (21, 23, 25). Therapeutic lifestyle interventions or medications for hypertension were started after completion of baseline examinations, including baPWV and echocardiography. Patients with the following conditions were excluded: (1) documented cardiovascular diseases, including coronary artery disease (history of coronary revascularization or myocardial infarction, or documented organic stenosis of ≥50% of epicardial coronary arteries on invasive angiography or computed tomography) or stroke, (2) history of cancer, secondary hypertension (functional or structural organic causes of hypertension, such as primary aldosteronism, pheochromocytoma, or renovascular hypertension), and other systemic diseases, including acute or chronic inflammatory diseases, (3) systolic blood pressure ≥180 mmHg and/or diastolic blood pressure ≥110 mmHg, (4) atrial fibrillation and uncontrolled arrhythmia, (5) valvular regurgitation or stenosis of greater than mild degree, (6) pericardial effusion, and (7) ankle-brachial index < 0.9 or > 1.4. The study complies with the Declaration of Helsinki, and the local institutional review board (IRB) of the study center approved the study protocol. Written informed consent was obtained from each study patient.

### Data Collection and Variable Definitions

Patients' clinical data were collected at the time of recruitment. Body mass index was calculated as the ratio of weight in kilograms divided by the height in meters squared (kg/m^2^). Obesity was defined as body mass index ≥25 kg/m^2^ according to the Asian-Pacific obesity criteria. Blood pressure and heart rate were measured after 5 min of rest. Mean arterial pressure was calculated by the following equation: (1 × systolic blood pressure + 2 × diastolic blood pressure)/3. Pulse pressure was calculated as the systolic blood pressure minus the diastolic blood pressure. Diabetes mellitus was defined by either a previous diagnosis of diabetes mellitus, the current use of oral antihyperglycemic agents or insulin, or fasting glucose ≥126 mg/ml or glycated hemoglobin ≥6.5%. Dyslipidemia was defined by one of the following: a previous diagnosis of dyslipidemia, lipid-lowering medications, or low-density lipoprotein cholesterol of ≥160 mg/dl. After 12 h or more of overnight fasting, we obtained venous blood levels of white blood cell, hemoglobin, glucose, glycated hemoglobin, creatinine, total cholesterol, low-density lipoprotein cholesterol, high-density lipoprotein cholesterol, triglyceride, and C-reactive protein. Glomerular filtration rate was calculated using the Modification of Diet in Renal Disease formula.

### Measurement of baPWV

All patients underwent baPWV measurement at the time of hypertension diagnosis, using an automated pulse waveform recorder (VP-1000; Colin Co. Ltd., Komaki, Japan) (12, 13, 15). In brief, patients were placed in the supine position after having 5 or more minutes of rest. On both the right and left sides, arterial pulse wave was measured at upper (brachial arteries) and lower (posterior tibial arteries) extremities, and baPWV was calculated as the distance between the brachial and posterior tibial arteries divided by the time interval between the two measurement points. The distance between the arteries was estimated based on the patient's height. Electrocardiogram, phonogram (cardiac auscultation), pulse volume waveform, blood pressure, and heart rate were recorded simultaneously during the measurements. The mean of right and left baPWV values was used for the study analysis. These measurements were made by the same experienced operator who was blinded to the patients' clinical information.

### Transthoracic Echocardiography

Transthoracic echocardiography was performed according to the contemporary guidelines (26), using a commercially available machine (Sequoia, Siemens Medical Solutions, CA, USA; or Vivid 9, GE Medical Systems, MA, USA). LV ejection fraction was calculated by the biplane Simpson's method. M-mode echocardiography was used to measure LV dimensions and wall thickness at the standard parasternal window. LV mass was calculated by a validated formula (26), and indexed to the body surface area (= LV mass index). The relative wall thickness of LV was calculated by the following equation: (2 × posterior wall thickness) / LV end-diastole diameter. LV hypertrophy was defined as LV mass index >115 g/m^2^ in men and >95 g/m^2^ in women, which was further categorized as concentric (relative wall thickness > 0.42) and eccentric (relative wall thickness ≤ 0.42) LV hypertrophy (27). Concentric remodeling was defined as a relative wall thickness > 0.42 in the absence of LV hypertrophy. Tissue Doppler velocities were obtained at the medial side of the mitral annulus. Early and late mitral inflow velocities were acquired at the tip level of the mitral valve from the apical 4-chamber view. Left atrial volume was calculated based on the measurements of apical 4- and 2-chamber views and also indexed to the body surface area. The presence of LV diastolic dysfunction was evaluated using four criteria (E/e′ ratio > 14, septal e′ velocity <7 cm/s, tricuspid regurgitant jet velocity > 2.8 m/s, and left atrial volume index > 34 ml/m^2^). A diagnosis of LV diastolic dysfunction was made if more than half of the criteria were met according to the 2016 American Society of Echocardiography/European Association of Cardiovascular Imaging guidelines (28).

### Statistical Analysis

Categorical variables are presented as frequencies (percentages) and continuous variables as mean ± standard deviation or median with interquartile range (IQR). The differences between the groups were analyzed using the χ^2^ test for categorical variables and Student's *t* test for continuous variables. Pearson's bivariate correlation analyses were performed to investigate the linear association between two continuous variables. Multivariable linear regression analysis was further performed with adjustment for known clinical risk factors: *model 1* included the covariates of age, sex, body mass index, systolic blood pressure, and blood levels of glucose, and low-density lipoprotein cholesterol; *model 2* included age, heart rate, smoking status, diabetes mellitus, glomerular filtration rate, and medication of statin. The relationship between baPWV and relevant variables were also depicted as scatterplots with linear regression lines with a 95% confidence interval. The receiver operating characteristic (ROC) curves analysis was performed to evaluate the discriminant ability of baPWV for predicting LV hypertrophy and diastolic dysfunction. The DeLong test was used to compare areas under the curve (AUC) of ROC. A two-tailed *P*-value of < 0.05 was considered statistically significant. All analyses were performed using R (version 3.6.0, Vienna, Austria).

## Results

### Clinical Characteristic of the Study Patients

Our study cohort comprised 202 patients diagnosed with hypertension, none of whom received previous antihypertensive medications. The baseline characteristics of these patients are summarized in Table 1. The mean age was 62.2 ± 11.9 years, and approximately half of them (48.5%) were women, with an elevated mean value of systolic blood pressure (150 ± 12 mmHg). The prevalence rates of diabetes mellitus, dyslipidemia, and obesity were 31.2, 30.7, and 45.0%, respectively. Twenty-six patients (12.9%) were current smokers. The mean values of fasting glucose, glycated hemoglobin, and C-reactive protein were mildly elevated, whereas cholesterol profiles were within normal limits. A small proportion of the patients were on the medication of statin (15.3%).

**Table 1:** Clinical characteristics of study patients.

**Characteristic**	**Value (*n* = 202)**
Age, years	62.2 ± 11.9
Female sex	98 (48.5)
Body mass index, kg/m^2^	25.2 ± 3.6
Systolic blood pressure, mmHg	150 ± 12
Diastolic blood pressure, mmHg	87.5 ± 9.6
Mean arterial pressure, mmHg	114 ± 10
Pulse pressure, mmHg	62.6 ± 11.8
Heart rate, per minute	70.0 ± 12.3
**Cardiovascular risk factors**	
Diabetes mellitus	63 (31.2)
Dyslipidemia	62 (30.7)
Current smoking	26 (12.9)
Obesity (body mass index ≥ 25 kg/m^2^)^*^	91 (45.0)
**Results of blood tests**	
White blood cell count, 10^3^ per μl	7.0 ± 2.5
Hemoglobin, g/dl	13.3 ± 1.9
Glucose, mg/dl	119 ± 34
Glycated hemoglobin, %	6.4 ± 1.3
Glomerular filtration rate, ml/min/1.73 m^2^	77.2 ± 30.6
Total cholesterol, mg/dl	165 ± 41
Low-density lipoprotein cholesterol, mg/dl	95.8 ± 34.9
High-density lipoprotein cholesterol, mg/dl	49.9 ± 13.5
Triglyceride, mg/dl	132 ± 85
C-reactive protein, mg/dl	1.2 ± 3.0
**Medications**	
Antiplatelets	13 (6.4)
Statin	31 (15.3)

*Numbers are expressed as n (%) or mean ± standard deviation*.

* *Asian-Pacific obesity criteria*.

Parameters of transthoracic echocardiography and baPWV are demonstrated in Table 2. LV ejection fraction was within the normal reference range. Approximately 40% of the patients had abnormal LV remodeling patterns, with the highest proportion of eccentric LV hypertrophy (24.3%). Concerning diastolic parameters, the mean values of septal e′ velocity and E/e′ were 6.0 ± 1.8 and 11.8 ± 3.6 cm/s, respectively. One-fourth of the patients (24.8%) had diastolic dysfunction defined by the 2016 American Society of Echocardiography/European Association of Cardiovascular Imaging guidelines (28). The mean of baPWV values from the entire study cohort was 1,748 ± 285 cm/s.

**Table 2:** Parameters of transthoracic echocardiography and baPWV of study patients.

**Characteristic**	**Value (*n* = 202)**
**Echocardiographic findings**	
LV ejection fraction, %	67.2 ± 5.4
Relative wall thickness	0.38 ± 0.05
LV mass index, g/m^2^	94.9 ± 22.8
Male	94.7 ± 24.3
Female	95.1 ± 21.2
LV remodeling patterns	
Normal	118 (58.4)
Concentric remodeling	14 (6.9)
Concentric LV hypertrophy	21 (10.4)
Eccentric LV hypertrophy	49 (24.3)
Septal e′ velocity, cm/s	6.0 ± 1.8
E/e′	11.8 ± 3.6
Left atrial volume index, mL/m^2^	32.6 ± 9.8
Maximal velocity of tricuspid regurgitation, m/s	2.4 ± 0.3
Diastolic dysfunction	50 (24.8)
Brachial-ankle pulse wave velocity, cm/s	1,748 ± 285

*Numbers are expressed as n (%) or mean ± standard deviation. baPWV, brachial-ankle pulse wave velocity; LV, left ventricular*.

### Associations of baPWV With the Indices of LV Geometry and Diastolic Function

**Figure 1:**
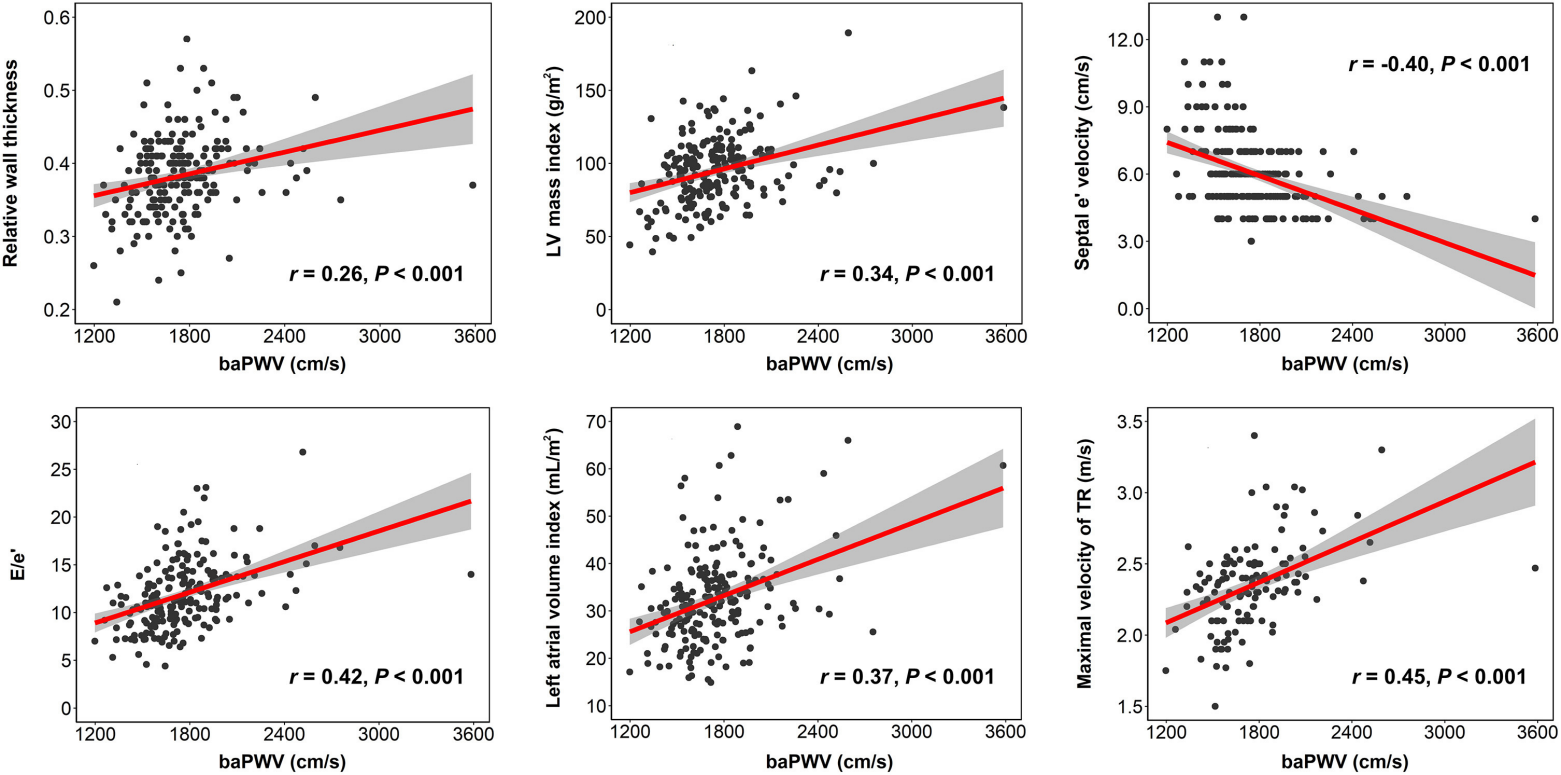
Correlation plots showing the associations of baPWV with indices of LV geometry and diastolic function. Each dot indicates an individual patient's data. Linear regression line (red line) and 95% confidence interval (shaded area) are depicted. baPWV, brachial-ankle pulse wave velocity; LV, left ventricular; TR, tricuspid regurgitation.

The result of simple correlations of baPWV with the indices of LV geometry and diastolic function are summarized in Table 3. The baPWV had significant positive correlations with structural measurements of LV, including relative wall thickness (*r* = 0.261, *P* < 0.001) and LV mass index (β = 0.339*, P* < 0.001). The baPWV also had significant correlations with all four diastolic parameters including septal e′ velocity (*r* = −0.400, *P* < 0.001), E/e′ (*r* = 0.424, *P* < 0.001), left atrial volume index (*r* = 0.369, *P* < 0.001), and maximal velocity of tricuspid regurgitation (*r* = 0.453, *P* < 0.001). Correlation plots between baPWV and these LV geometry and diastolic parameters are demonstrated in Figure 1. When adjusted for multiple clinical risk factors, these associations remained significant in the multivariable linear regression analyses (*P* < 0.05 for each in both *model 1* and *model 2*) (Table 4).

**Table 3:** Simple correlations of baPWV with indices of LV geometry and diastolic function.

**Parameter**	***r***	***P***
Relative wall thickness	0.261	<0.001
LV mass index	0.339	<0.001
Septal e′ velocity	−0.400	<0.001
E/e′	0.424	<0.001
Left atrial volume index	0.369	<0.001
Maximal velocity of tricuspid regurgitation	0.453	<0.001

*baPWV, brachial-ankle pulse wave velocity; LV, left ventricular*.

**Table 4:** Multiple linear regression analysis showing the associations of baPWV with parameters of LV geometry and diastolic function.

	**Model 1**		**Model 2**	
**Parameter**	**β**	***P***	**β**	***P***
Relative wall thickness	0.219	0.021	0.226	0.008
LV mass index	0.286	0.002	0.331	<0.001
Septal e′ velocity	−0.199	0.018	−0.249	0.001
E/e′	0.373	<0.001	0.206	0.005
Left atrial volume index	0.334	<0.001	0.338	<0.001
Maximal velocity of tricuspid regurgitation	0.401	<0.001	0.377	<0.001

*Model 1 was adjusted for age, sex, body mass index, systolic blood pressure, glucose and low-density lipoprotein cholesterol. Model 2 was adjusted for age, heart rate, smoking status, diabetes mellitus, glomerular filtration rate, and medication of statin. baPWV, brachial-ankle pulse wave velocity; LV, left ventricular*.

### baPWV Values According to LV Remodeling Patterns and Diastolic Dysfunction

**Figure 2:**
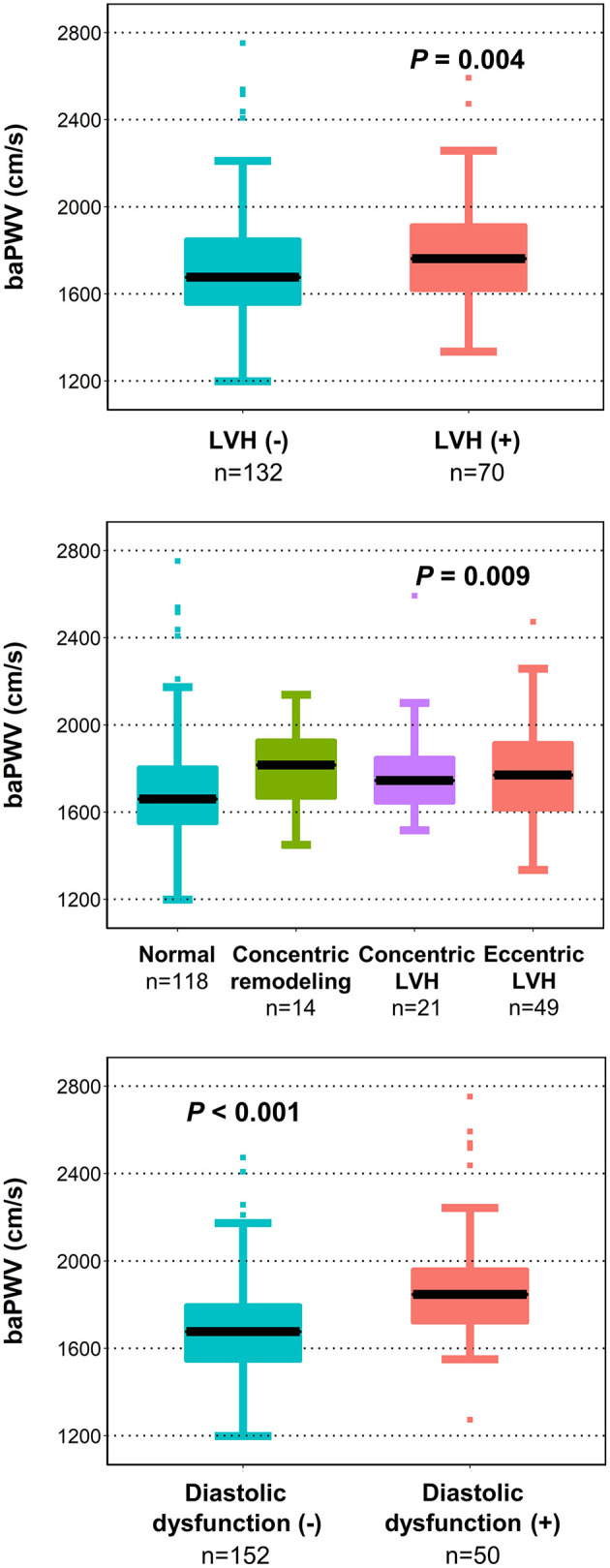
Association of LV remodeling patterns and diastolic dysfunction with baPWV. The baPWV value by LV remodeling patterns and diastolic dysfunction is shown as a boxplot with the median (black line) and interquartile range. baPWV, brachial-ankle pulse wave velocity; LV, left ventricular; LVH, left ventricular hypertrophy.

We further examined whether arterial stiffness is associated with adverse LV structure and diastolic dysfunction (Figure 2). Patients with LV hypertrophy had significantly higher baPWV values compared to those without [1762 cm/s (IQR, 1,619 cm/s−1,920 cm/s) vs. 1,677 cm/s (IQR, 1,554 cm/s−1,852 cm/s), *P* = 0.004]. When further stratified into four groups of LV hypertrophic remodeling patterns, the group of patients with concentric remodeling as well as that with concentric and eccentric LV hypertrophy had elevated baPWV compared to patients with normal LV (*P* = 0.009). Patients with diastolic dysfunction also had higher baPWV values compared to those without [1,847 cm/s (IQR, 1,719 cm/s−1,962 cm/s) vs. 1,677 cm/s (IQR, 1,544 cm/s−1,800 cm/s), *P* < 0.001].

### Discriminant Ability of baPWV for Predicting LV Hypertrophy and Diastolic Dysfunction

**Figure 3:**
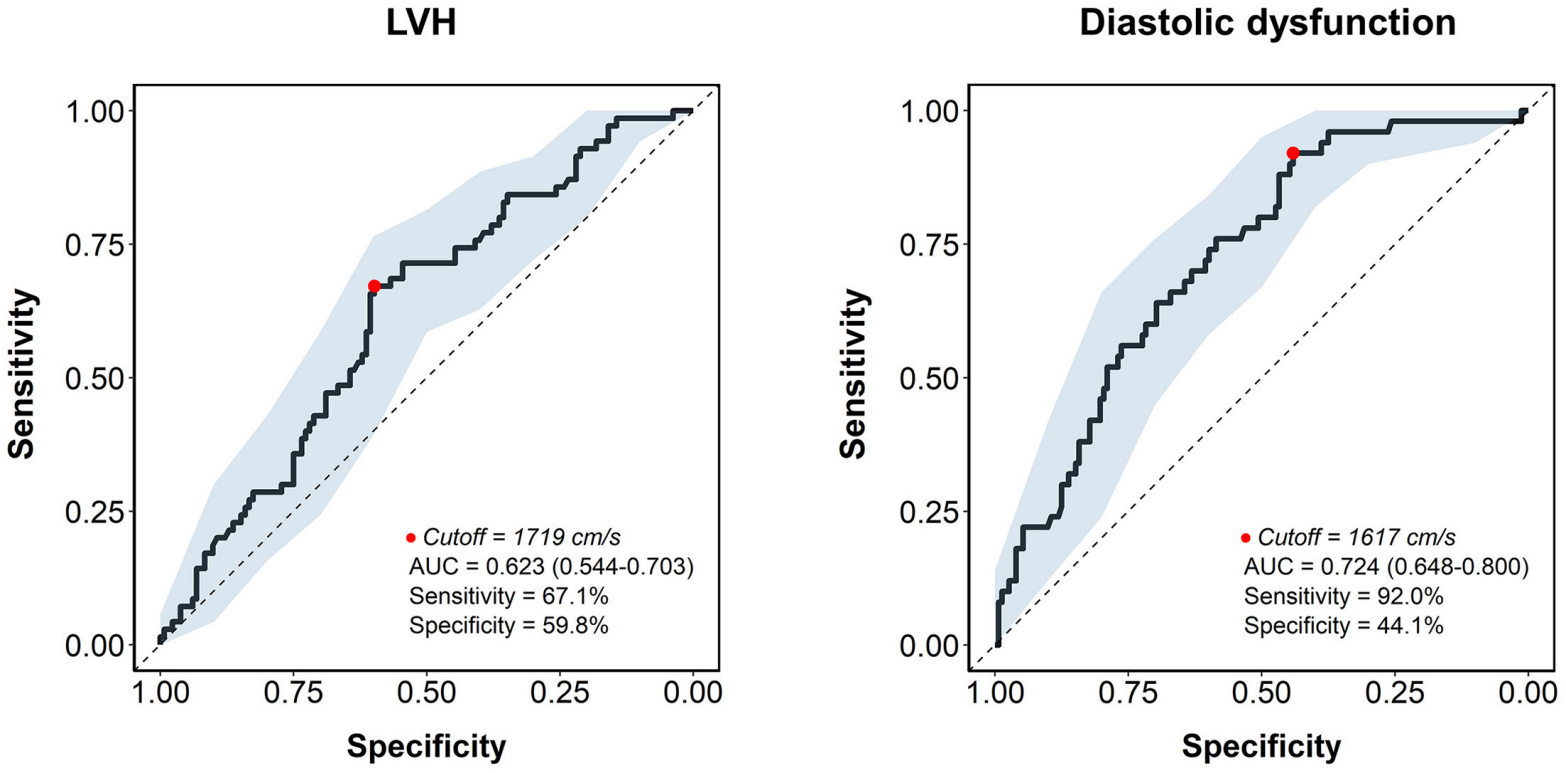
The receiver operating characteristic curve analysis of baPWV for predicting LV hypertrophy and diastolic dysfunction. The receiver operating characteristic curve is depicted as a solid line with 95% confidence interval (shaded area). The cutoff point is marked with a red dot. AUC, area under the curve; baPWV, brachial-ankle pulse wave velocity; LVH, left ventricular hypertrophy.

In the ROC analysis, the AUC of baPWV for the prediction of LV hypertrophy was 0.623 [95% confidence interval (CI), 0.544–0.703, *P* = 0.004], with 67.1% sensitivity and 59.8% specificity for a best cutoff value of 1,719 cm/s (Figure 3). The AUC was larger for predicting diastolic dysfunction with baPWV (AUC, 0.724, 95% CI, 0.648–0.800, *P* < 0.001), which had the cutoff value of 1,617 cm/s with 92.0% sensitivity and 44.1% specificity (Figure 3). The discriminant ability of the baPWV was similar to the pulse pressure in predicting LV hypertrophy (AUC, 0.623 vs. 0.624, *P* = 0.975). The baPWV had a higher AUC for predicting diastolic dysfunction compared to that of the pulse pressure, although not statistically significant (AUC, 0.724 vs. 0.698, *P* = 0.566).

### Sex-Specific Associations of baPWV With LV Geometry and Diastolic Parameters

In our cohort, women were older than men (65.0 ± 9.9 years vs. 59.5 ± 12.9 years, *P* = 0.001), with more frequent LV hypertrophy and elevated E/e' (*P* < 0.001 for each) (Supplementary Table 1). Women had higher baPWV compared to men (1788 ± 326 cm/s vs. 1,711 ± 235 cm/s, *P* = 0.056).

The simple correlations between baPWV and the parameters of LV geometry and diastolic function were significant in both sex (Supplementary Table 2), as in the entire cohort. In the multivariable analysis, these associations were consistently observed in both men and women, except for the relative wall thickness not significant within women in both adjustment models (Supplementary Table 2). Within each sex, baPWV was significantly elevated in patients with LV hypertrophy, abnormal LV structural, and diastolic dysfunction than those without (Supplementary Figure 1). In the ROC analysis, the baPWV showed a fair discrimination ability for predicting diastolic dysfunction in both sex (Supplementary Figure 2).

## Discussion

This study demonstrated that the baPWV had significant associations with relative wall thickness, LV mass index, and four diastolic parameters in untreated hypertensive patients, even after controlling for multiple confounding factors. The baPWV was significantly elevated with patients with LV hypertrophy, abnormal LV remodeling, and diastolic dysfunction and also showed fair discrimination ability, especially for predicting diastolic dysfunction. The result of this study provides additional evidence for a significant contribution of arterial stiffness to LV remodeling and diastolic function. Furthermore, our results highlight the clinical value of baPWV in risk stratification and an initial screening test for newly diagnosed hypertensive patients.

Hypertension is highly prevalent and a powerful contributor to fatal cardiovascular events. The key step in managing hypertensive patients is assessing the degree and extent of hypertensive-mediated organ damage (HMOD), which are the subclinical structural and functional end-organ changes under the long-standing exposure to high blood pressure (21, 23, 25). Cardiovascular risk is significantly increased with the presence of HMOD such as LV hypertrophy or carotid intima-media wall thickening (21, 25). Furthermore, the risk becomes even higher when HMOD affects multiple organs (29). Therefore, the guidelines strongly recommend the routine assessment of HMOD in all hypertensive patients for deciding the initiation of antihypertensive medications, selecting specific drugs, and setting a blood pressure therapeutic target (21, 23, 25).

Multiple lines of evidence suggest that PWV has substantial value in predicting adverse cardiovascular events. A meta-analysis of 17,635 subjects involving the general population or patients with cardiovascular risk factors demonstrated that PWV is significantly associated with the incidence of coronary heart disease, stroke, and cardiovascular mortality, with an approximately 100 cm/s increase in cfPWV corresponding to a 7% increased risk (3). This was also confirmed in a more recent meta-analysis from the Japanese population (7). The prognostic value of PWV was also consistent among the untreated hypertensive patients, and a previous prospective cohort study reported that baPWV ≥ 1,750 cm/s was associated with a 3-fold increased risk of cardiovascular events within the patients (8). In addition, higher PWV was independently related to a greater burden of cardiac damage in untreated hypertensive patients, including decreased coronary flow reserve and left atrial enlargement (30), suggesting its role as an initial screening tool in hypertension.

However, the routine use of PWV for estimating the extent of organ damage in hypertensive patients is not well-established. While the European guideline suggested cfPWV as an optional assessment for HMOD (21), the American guideline had no specific recommendation for any use of PWV in hypertensive subjects (25). In the Japanese guideline, on the other hand, both cfPWV and baPWV are available examinations for detecting vascular damage (23). The limited use of PWV across the guidelines may be partially attributed to methodological difficulties in measuring cfPWV. Although cfPWV is considered the standard method, it requires technical precision to accurately record carotid waveform and femoral artery pulse measurement, which causes significant inconvenience and stress to patients, discouraging its use on a regular basis in practice (20, 22, 24). In contrast, baPWV is a more simplified and easier method, which can be obtained solely by wrapping pressure cuffs of upper arms and ankles. Regarding the capability of HMODs assessment, a study from northern Shanghai reported that cfPWV may be more closely related to the creatinine clearance and carotid artery intimal-media thickness compared to the baPWV, but there was no significant difference observed for the association with cardiac damages between the two methods, including LV mass index or E/e' (11). Other studies also showed that the baPWV has significant associations with HMODs similar to the cfPWV (31, 32), suggesting baPWV as an acceptable alternative to the cfPWV. Therefore, baPWV may hold promise as an initial screening test for better risk stratification in hypertensive patients.

Our study demonstrated that baPWV is significantly associated with the indices of LV remodeling and diastolic parameters in treatment-naïve hypertensive patients. Although many studies investigated the association of baPWV with cardiac damages previously, the majority of them included patients who are already treated with antihypertensive medications or a mixed population with and without hypertension (11–19), making it difficult to establish its role as an initial screening test. We here provide evidence from the well-defined cohort of previously untreated hypertensive patients to examine such role of baPWV. Indeed, the discriminant ability of baPWV for predicting LV hypertrophy and diastolic dysfunction was significant, with a more fair discrimination function for detecting diastolic dysfunction (Figure 3). Of note, the associations of baPWV with echocardiography parameters and predictability of baPWV was generally consistent in each group of men and women, suggesting it may be a useful screening tool in both sexes.

Chronic pressure overload in hypertension induces various remodeling of the heart (33). The primary response is the hypertrophic growth of cardiomyocytes, resulting in the thickening of the LV wall. Myocardial fibrosis also develops and contributes to increased LV stiffness, which is thought to play a key role in diastolic dysfunction (33). Moreover, hypertension increases shear stress on the endothelial wall and promotes coronary microvascular damage (34, 35). As an early marker of myocardial injury, coronary microvascular damage is an important pathophysiology of ischemic heart disease and is also closely linked to the presence of diastolic dysfunction, even without obstructive coronary artery disease (34, 35). These features are well-established prognosticators for poor cardiovascular outcomes among hypertensive patients (36, 37). Importantly, reduction in blood pressure prevent the progression of LV hypertrophy and not infrequently regress LV mass (36). This effect leads to improved prognosis for hypertensive patients and may reflect the appropriate treatment response (38). Several studies also suggested that early blood pressure control may restore diastolic dysfunction in hypertension (37, 39). Therefore, the timely evaluation of baPWV may provide valuable information when estimating cardiovascular risks and related cardiac damage among hypertensive patients (40–42).

Several mechanisms underlying the associations of arterial stiffness with LV remodeling and diastolic function have been suggested. The most plausible explanation is that stiffened arteries reflect the pressure pulse wave more rapidly backward to LV, resulting in increased afterload of LV (13–16, 43). This could cause LV hypertrophy and a reduction in coronary blood flow during the diastole, which are thought to be the main contributors to impaired LV relaxation. This hypothesis is further supported by the significant associations between arterial stiffness and LV stiffness (43, 44).

## Study Limitations

This study has some limitations. First, this study could not provide casual relationships of baPWV with LV geometry and diastolic parameters due to its cross-sectional design. The impact of increased baPWV on the myocardial damages for the long-term follow up period could be explored in future studies with the longitudinal design. In addition, since the patients enrolled were all Koreans, it should be further investigated whether this result could be generalized to other ethnicities.

## Conclusion

In treatment-naive hypertensive patients, baPWV is significantly associated with LV geometry and diastolic dysfunction. Because the measurement of baPWV is very simple, it may be useful in the initial examination of hypertensive patients.

## Data Availability Statement

The raw data supporting the conclusions of this article will be made available by the authors, without undue reservation.

## Ethics Statement

The studies involving human participants were reviewed and approved by Boramae Medical Center, Seoul; IRB number: 26-2017-22. The patients/participants provided their written informed consent to participate in this study.

## Author Contributions

SK and MI performed the statistical analysis. SK drafted the manuscript. H-LK, MI, W-HL, J-BS, S-HK, J-HZ, and M-AK reviewed/edited the manuscript and contributed to the interpretation of data. H-LK conceptualized the overall study design and supervised all aspects of the study and revised the manuscript critically. All authors have read and approved the manuscript.

## Conflict of Interest

The authors declare that the research was conducted in the absence of any commercial or financial relationships that could be construed as a potential conflict of interest.

## Abbreviations

AUC: area under the curve
baPWV: brachial-ankle pulse wave velocity
cfPWV: brachial-ankle pulse wave velocity
HMOD: hypertension mediated organ damage
IQR: interquartile range
LV: left ventricle
PWV: pulse wave velocity
ROC: receiver operating characteristic.
