# Supplementary material for: Associations of Brachial-Ankle Pulse Wave Velocity With Left Ventricular Geometry and Diastolic Function in Untreated Hypertensive Patients
Source: Front Cardiovasc Med. 2021 May 10;8:647491. doi: 10.3389/fcvm.2021.647491 (PMC8143267; doi:10.3389/fcvm.2021.647491)

**SUPPLEMENTAL MATERIALS**

**Supplemental Table 1. Comparison of clinical characteristics between men and women.**

| **Characteristic** | **Men (n=104)** | **Women (n=98)** | ***P*** |
| --- | --- | --- | --- |
| Age, years | 59.5 ± 12.9 | 65.0 ± 9.9 | 0.001 |
| Body mass index, kg/m^2^ | 25.6 ± 3.4 | 24.9 ± 3.7 | 0.156 |
| Systolic blood pressure, mmHg | 149.2 ± 11.4 | 151.0 ± 12.5 | 0.273 |
| Diastolic blood pressure, mmHg | 88.8 ± 9.9 | 86.1 ± 9.1 | 0.048 |
| Mean arterial pressure, mmHg | 113.8 ± 9.9 | 114.7 ± 9.5 | 0.509 |
| Pulse pressure, mmHg | 60.4 ± 11.0 | 64.9 ± 12.3 | 0.007 |
| Heart rate, per minute | 70.4 ± 12.5 | 69.5 ± 12.3 | 0.604 |
| *Cardiovascular risk factors* |  |  |  |
| Diabetes mellitus | 34 (32.7) | 29 (29.6) | 0.746 |
| Dyslipidemia | 22 (21.2) | 40 (40.8) | 0.004 |
| Current smoking | 25 (24.0) | 1 (1.0) | < 0.001 |
| Obesity (body mass index ≥25 kg/m^2^)* | 55 (52.9) | 36 (36.7) | 0.030 |
| *Results of blood tests* |  |  |  |
| White blood cell count, 10^3^ per *μ*L | 7.3 ± 2.8 | 6.8 ± 2.2 | 0.152 |
| Hemoglobin, g/dL | 13.5 ± 2.1 | 13.0 ± 1.5 | 0.097 |
| Glucose, mg/dL | 124.1 ± 40.7 | 114.3 ± 25.4 | 0.053 |
| Glycated hemoglobin, % | 6.6 ± 1.5 | 6.3 ± 1.1 | 0.159 |
| Glomerular filtration rate, mL/min/1.73m^2^ | 76.2 ± 34.5 | 78.3 ± 25.8 | 0.634 |
| Total cholesterol, mg/dL | 164.7 ± 44.5 | 165.7 ± 38.1 | 0.865 |
| Low-density lipoprotein cholesterol, mg/dL | 95.6 ± 36.7 | 96.0 ± 33.3 | 0.952 |
| High-density lipoprotein cholesterol, mg/dL | 47.8 ± 13.6 | 52.0 ± 13.1 | 0.043 |
| Triglyceride, mg/dL | 137.0 ± 93.3 | 126.0 ± 76.1 | 0.406 |
| C-reactive protein, mg/dL | 1.4 ± 3.4 | 1.1 ± 2.5 | 0.556 |
| *Medications* |  |  |  |
| Antiplatelets | 6 (5.8) | 7 ( 7.1) | 0.912 |
| Statin | 12 (11.5) | 19 (19.4) | 0.177 |
| *Echocardiographic findings* |  |  |  |
| LV ejection fraction, % | 67.1 ± 5.5 | 67.3 ± 5.2 | 0.849 |
| Relative wall thickness | 0.4 ± 0.1 | 0.4 ± 0.1 | 0.470 |
| LV mass index, g/m^2^ | 94.7 ± 24.3 | 95.1 ± 21.2 | 0.891 |
| LV remodeling patterns |  |  | < 0.001 |
| Normal | 74 (71.2) | 44 (44.9) |  |
| Concentric remodeling | 10 (9.6) | 4 (4.1) |  |
| Concentric LV hypertrophy | 7 (6.7) | 14 (14.3) |  |
| Eccentric LV hypertrophy | 13 (12.5) | 36 (36.7) |  |
| Septal e′ velocity, m/s | 0.06 ± 0.02 | 0.06 ± 0.02 | 0.014 |
| E/e′ | 11.0 ± 3.3 | 12.7 ± 3.7 | < 0.001 |
| Left atrial volume index, mL/m^2^ | 31.5 ± 9.2 | 33.8 ± 10.4 | 0.098 |
| Maximal velocity of  tricuspid regurgitation, m/s | 2.3 ± 0.3 | 2.4 ± 0.3 | 0.610 |
| Diastolic dysfunction | 22 (21.2) | 28 (28.6) | 0.290 |
| Brachial-ankle pulse wave velocity, cm/s | 1711 ± 235 | 1788 ± 326 | 0.056 |

Numbers are expressed as n (%) or mean ± standard deviation. LV, left ventricular.

*Asian-Pacific obesity criteria.

**Supplemental Table 2. Simple and multiple linear regression analysis of baPWV with parameters of LV geometry and diastolic function according to sex.**

| **Parameter** | **Men (n=104)** | | **Women (n=98)** | |
| --- | --- | --- | --- | --- |
|  | ***β*** | ***P*** | ***β*** | ***P*** |
| ***Simple correlation*** |  |  |  |  |
| Relative wall thickness | 0.267 | 0.006 | 0.255 | 0.011 |
| LV mass index | 0.279 | 0.004 | 0.412 | < 0.001 |
| Septal e′ velocity | -0.351 | < 0.001 | -0.443 | < 0.001 |
| E/e′ | 0.415 | < 0.001 | 0.406 | < 0.001 |
| Left atrial volume index | 0.289 | 0.003 | 0.410 | < 0.001 |
| Maximal velocity of  tricuspid regurgitation | 0.416 | < 0.001 | 0.492 | < 0.001 |
| ***Multiple linear regression*** |  |  |  |  |
| ***Model 1*** |  |  |  |  |
| Relative wall thickness | 0.271 | 0.037 | 0.147 | 0.298 |
| LV mass index | 0.264 | 0.041 | 0.269 | 0.048 |
| Septal e′ velocity | -0.207 | 0.076 | -0.206 | 0.089 |
| E/e′ | 0.445 | < 0.001 | 0.307 | 0.017 |
| Left atrial volume index | 0.274 | 0.032 | 0.360 | 0.007 |
| Maximal velocity of  tricuspid regurgitation | 0.396 | 0.019 | 0.455 | 0.002 |
| ***Model 2*** |  |  |  |  |
| Relative wall thickness | 0.294 | 0.013 | 0.143 | 0.249 |
| LV mass index | 0.278 | 0.017 | 0.378 | 0.002 |
| Septal e′ velocity | -0.229 | 0.039 | -0.210 | 0.041 |
| E/e′ | 0.224 | 0.025 | 0.123 | 0.250 |
| Left atrial volume index | 0.224 | 0.053 | 0.436 | < 0.001 |
| Maximal velocity of  tricuspid regurgitation | 0.350 | 0.013 | 0.371 | 0.008 |

Model 1 was adjusted for age, body mass index, systolic blood pressure, glucose, and low-density lipoprotein cholesterol. Model 2 was adjusted for age, heart rate, smoking status, diabetes mellitus, glomerular filtration rate, and medication of statin. baPWV, brachial-ankle pulse wave velocity; LV, left ventricular.

**Supplemental Figure 1. Association of LV remodeling patterns and diastolic dysfunction with baPWV according to sex.**

baPWV, brachial-ankle pulse wave velocity; LV, left ventricular; LVH, left ventricular hypertrophy.


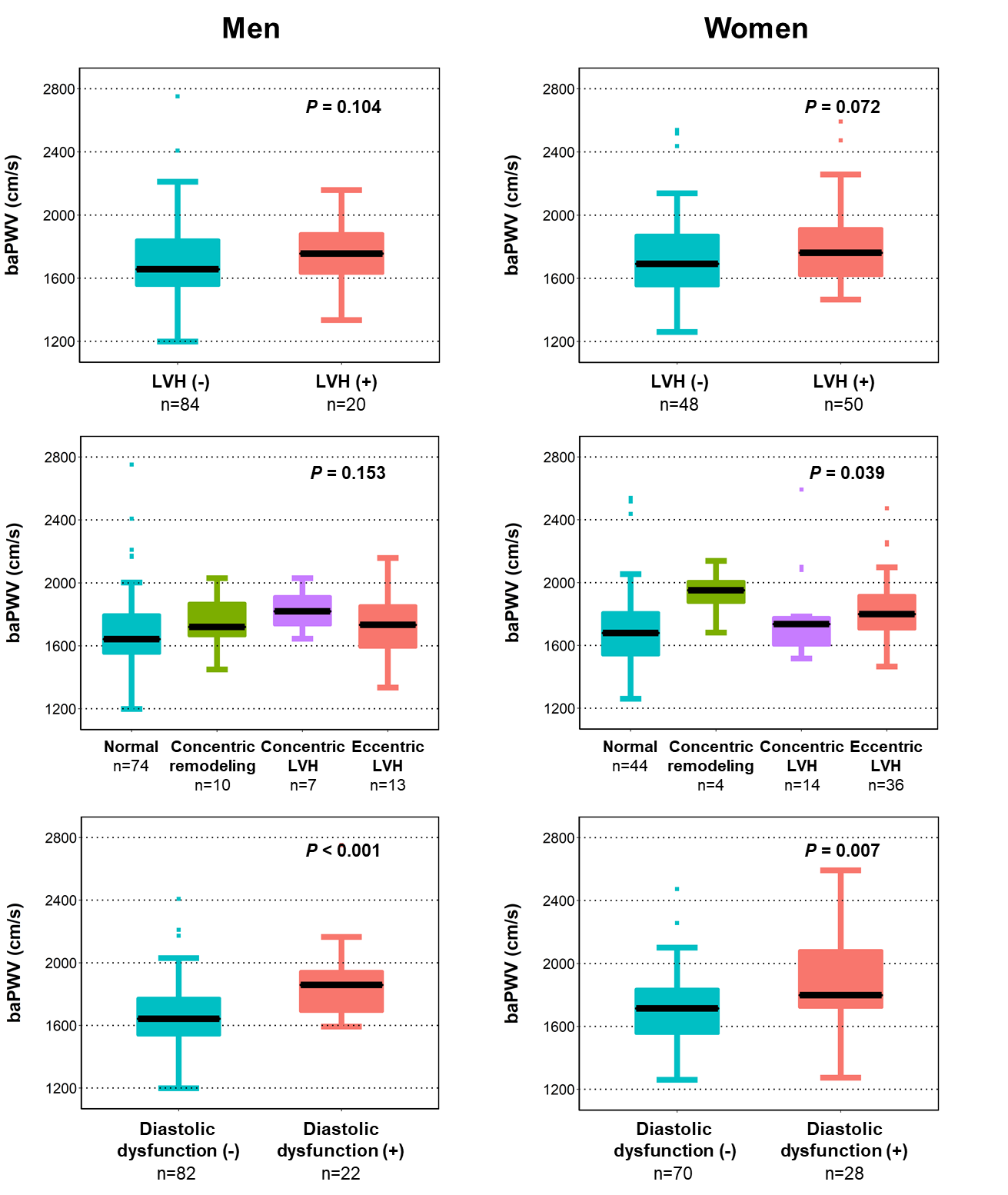


**Supplemental Figure 2. The receiver operating characteristic curve analysis of baPWV for predicting LV hypertrophy and diastolic dysfunction according to sex.**

AUC, area under the curve; baPWV, brachial-ankle pulse wave velocity; LVH, left ventricular hypertrophy.


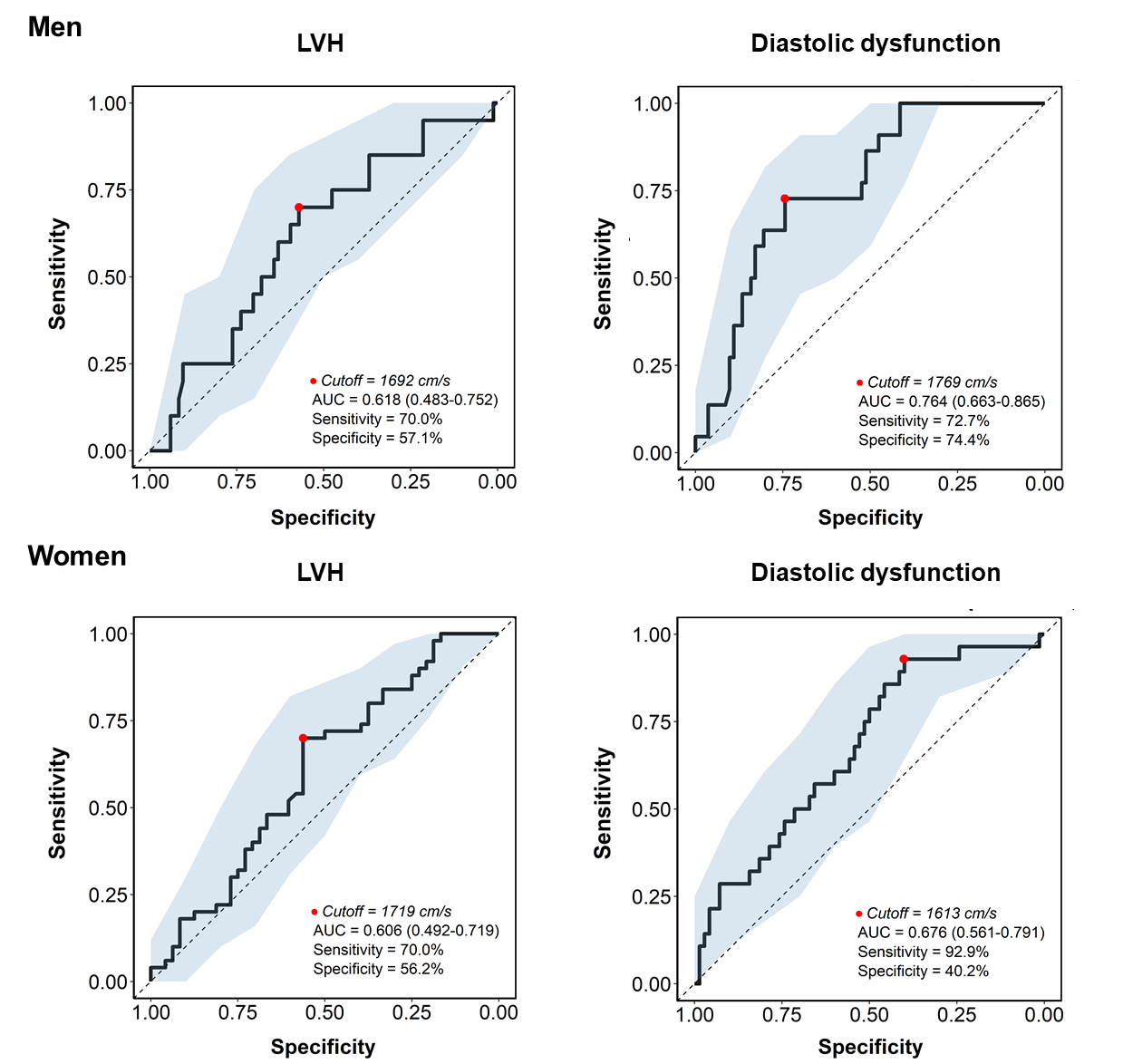

Supplement: Supplementary file 1 [file Data_Sheet_1.docx]
